# HIV-1 immunogens and strategies to drive antibody responses towards neutralization breadth

**DOI:** 10.1186/s12977-018-0457-7

**Published:** 2018-11-26

**Authors:** Jelle van Schooten, Marit J. van Gils

**Affiliations:** Department of Medical Microbiology, Amsterdam UMC, University of Amsterdam, Location AMC, Meibergdreef 9, Room K3-105, 1105AZ Amsterdam, The Netherlands

## Abstract

Despite enormous efforts no HIV-1 vaccine has been developed that elicits broadly neutralizing antibodies (bNAbs) to protect against infection to date. The high antigenic diversity and dense *N*-linked glycan armor, which covers nearly the entire HIV-1 envelope protein (Env), are major roadblocks for the development of bNAbs by vaccination. In addition, the naive human antibody repertoire features a low frequency of exceptionally long heavy chain complementary determining regions (CDRH3s), which is a typical characteristic that many HIV-1 bNAbs use to penetrate the glycan armor. Native-like Env trimer immunogens can induce potent but strain-specific neutralizing antibody responses in animal models but how to overcome the many obstacles towards the development of bNAbs remains a challenge. Here, we review recent HIV-1 Env immunization studies and discuss strategies to guide strain-specific antibody responses towards neutralization breadth.

## Introduction

The human immunodeficiency virus type 1 (HIV-1) continues to be a global health threat and the development of an effective vaccine is highly desirable to eradicate HIV/AIDS. Most existing viral vaccines work through the induction of neutralizing antibodies (NAbs) that block infection and/or viremia [1]. Despite enormous scientific progress since the discovery of HIV-1 as the causative agent of AIDS in the early 1980s, a safe and effective vaccine remains elusive. One of the greatest challenges of developing an effective vaccine with broad coverage is the extraordinary degree of genetic diversity within HIV-1 around the world and more specifically the high sequence variability in the HIV-1 envelope glycoprotein (Env) [2, 3], the sole target for NAbs. Viruses of the HIV-1 group M are responsible for the current HIV/AIDS pandemic and exhibit great viral diversity in their *env* gene [2, 3]. The clades of group M can differ by 25–35% in amino acid composition in Env, whereas variation within a clade is usually 15–20% [2–4]. Furthermore, Env contains many co-translationally attached *N*-linked glycans which shield the underlying protein surface from NAbs [5, 6]. In HIV-1 infected individuals, NAbs are produced against the Env of the transmitted/founder (T/F) virus within weeks to months after primary infection [7–11]. This initial antibody response is primarily strain-specific (autologous) and unable to cross-neutralize other genetically diverse heterologous HIV-1 isolates [8, 10–14]. The selective pressure exerted by the autologous antibody response drives viral evolution in HIV-1 infected individuals and causes the virus to escape from neutralization [7, 8, 14–19]. After several years of infection, in approximately 20–30% of HIV-1 infected individuals the autologous antibody response develops the ability to cross-neutralize a broad range of different (heterologous) viral strains [9, 12, 13, 20–23]. Moreover, one study even reports that sera from 50% of 205 chronic HIV-1 infected individuals were able to neutralize a wide panel of heterologous HIV-1 virus isolates [24]. This suggests that almost half of all HIV-1 infected individuals are capable of producing NAb responses with moderate to strong NAb breadth and potency. These NAb responses are likely to involve multiple monoclonal antibodies (mAbs) and maybe different specificities that together achieve broad serum neutralization. Studying HIV-1 specific NAb responses has led to the isolation and identification of broadly neutralizing antibodies (bNAbs), which have remarkable potency and cross-neutralizing activity against diverse HIV-1 clades worldwide, mostly by targeting highly conserved sites on Env, such as the CD4-binding site (CD4bs). Passive transfer studies with bNAbs in non-human primates have demonstrated effective protection against simian/human immunodeficiency virus (SHIV) [25–27]. Therefore, it is widely believed that vaccine-elicited bNAbs would effectively protect against HIV-1 infection in humans, but no vaccine has been developed that is able to do so. Here, we will review the recent developments of NAb responses induced by HIV-1 Env immunogens and discuss immunization strategies to drive the antibody response towards neutralization breadth.

## Vaccine-elicited autologous neutralizing antibody responses

HIV-1 isolates are widely used to determine the ability of vaccine-elicited antibody responses to neutralize HIV-1 virus variants in vivo. These HIV-1 isolates can be categorized based on their neutralization sensitivity ‘Tier’ phenotype [28, 29], with Tier-1 viruses being highly sensitive to antibody-mediated neutralization. Tier-1 viruses include lab-adapted viruses and a small fraction of circulating strains, whereas Tier-2 viruses have a higher resistance profile and comprise the majority of circulating HIV-1 strains.

As Env is the only target for NAbs, a wide range of different Env immunogens have been developed to induce NAbs against HIV-1. A synthetic peptide consisting of a part of the V3 region of gp120 (subunit of HIV-1 Env) was among the first immunogens able to induce a NAb response against a Tier-1 virus in rhesus monkeys [30]. Since then, a tremendous amount of immunization studies have been conducted using various Env immunogens including peptides, epitope scaffolds, recombinant gp120 proteins and uncleaved Envs [31]. However, these immunogens primarily elicited NAb responses against Tier-1 viruses and only sporadically against more neutralization-resistant circulating Tier-2 viruses [32–44]. Most of the Env immunogens tested so far did not resemble the native Env structure and therefore elicited mAbs against epitopes which are not well exposed on primary Tier-2 isolates or are shielded by the quaternary structure of the viral Env spike [28, 45–48]. Moreover, electron microscopy studies have demonstrated that non-native Env immunogens elicit mAbs against the CD4bs with an angle of approach that prevents binding to native Env trimer [49]. In recent years, native-like trimers based on the SOSIP design and related designs have consistently induced autologous Tier-2 NAb responses in different animal models [50–56]. These native-like trimers differ from other non-native Env immunogens as they mimic the structure of Env on the surface of the virus correctly and display multiple bNAb epitopes in a similar manner as how these epitopes appear on the viron-associated spike. Nevertheless, these native-like HIV-1 Env immunogens are not yet able to induce strong bNAb responses.

To better understand the limited breadth of vaccine-elicited NAbs, elaborate serological analyses have been performed and demonstrate that immunodominant strain-specific glycan holes on Env have been responsible for the narrow breadth of these mAbs [36, 43, 57–59]. The immunodominance of a breach in the glycan shield was first observed in immunization studies with virus-like particles (VLPs) expressing JR-FL Env trimers where NAb responses targeted a site lacking a highly conserved glycan at residue 197 [43]. The immunodominance of a glycan hole was also confirmed in immunization studies with native-like trimers based on the viral isolate BG505, termed BG505 SOSIP trimers [50, 58–62], through serum neutralization analysis and the isolation of mAbs [58]. Whether NAbs against strain-specific glycan holes can be broadened and eventually can develop into bNAbs remains to be determined.

## Vaccine-elicited heterologous neutralizing antibody responses

Much can be learned from the few instances of Env induced heterologous NAbs in animal models. Understanding the factors that caused the heterologous response in these few animals might help develop strategies to drive NAb responses towards breadth in the majority of vaccinees. In some species such as cows and llamas, bNAbs against HIV-1 have been elicited by vaccination [63–65]. From llamas immunized with a mixture of recombinant trimeric gp140s from clades A and BC, heavy chain-only antibodies were isolated with broad and potent neutralizing activity targeting the CD4bs [64, 65]. Llamas produce heavy chain-only antibodies which are much smaller in size than conventional mAbs and can therefore contact regions on Env that are normally inaccessible for human mAbs because of the glycan shield and/or other steric constraints. In another study, cows immunized with BG505 SOSIP trimers rapidly developed broad and potent serum HIV-1 specific NAb responses [63]. The BG505 SOSIP trimer has failed, however, to elicit bNAbs in other animal models with repertoires that resemble the human antibody repertoire more closely [50, 54]. Isolation and characterization of NAbs from these cows revealed that these NAbs contained ultralong third heavy chain complementary determining regions (CDRH3s) able to reach the CD4bs through the glycan shield. Cows generally have very long CDRH3s in their antibody repertoire with an average CDRH3 length of 26 amino acids and up to more than 70 amino acids [63, 66], whereas the average CDRH3 length in humans is approximately 13–15 amino acids [67, 68]. Very long CDRH3s are also a typical characteristic of human bNAbs and often necessary to penetrate the dense glycan shield. Thus, despite the glycan shield of HIV-1 Env, bNAbs can be elicited in cows and llamas because of the specific nature of their naive antibody repertoire. However, long CDRH3s are rare in the human antibody repertoire [69] and as a consequence fewer antibody precursors with the capacity to develop into bNAbs are present, making it less likely that humans will develop similar responses as observed in cows and llamas, unless innovative immunogen design strategies are developed to select for these rare precursors.

## Strategies to elicit heterologous NAb responses through vaccination

**Fig. 1:**
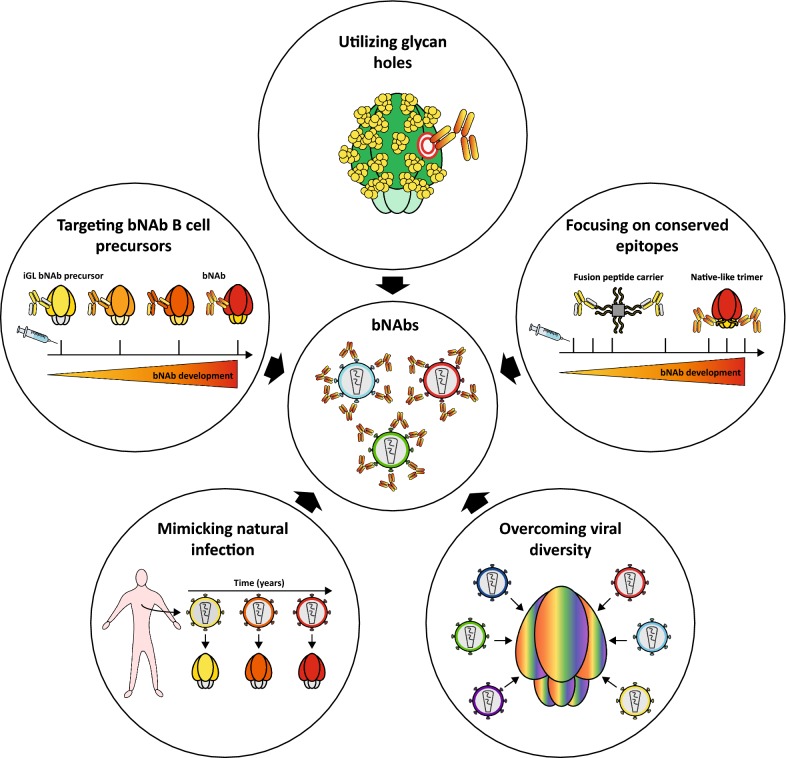
Strategies to induce broadly neutralizing antibodies. A schematic overview of various HIV-1 immunogen design strategies to drive autologous neutralizing antibody responses (NAb) towards the development of broadly neutralizing antibodies (bNAbs). Various immunogen strategies are currently exploited to drive the autologous NAb responses towards neutralization breadth and consist of (i) the selective removal of glycans to focus antibody responses towards a specific site of interest such as the CD4bs (ii) immunogens to guide the antibody response towards conserved sites such as the fusion peptide (iii) mosaic or consensus antigens to overcome the viral diversity of the circulating HIV-1 viruses worldwide (iv) lineage immunogens resembling virus evolution in HIV-1 infected elite neutralizers to recapitulate natural infection (v) immunogens targeting the inferred germline (iGL) precursors of bNAbs followed by further immunizations to guide the affinity maturation pathway towards the development of bNAbs. Importantly, one HIV-1 immunogen design strategy does not exclude the others and to efficiently induce bNAbs by vaccination, the integration of all different strategies into one coordinated vaccination regime is most likely required

To date, only a few immunogens have sporadically induced cross-NAbs against more neutralization-resistant Tier-2 viruses in commonly used animal models, such as guinea pigs, rabbits and macaques although these responses were generally weak and only occurred in very few animals [54, 70–79]. With the development of native-like Env trimer proteins, autologous Tier-2 responses are now consistently induced in animal models, but these neutralizing responses primarily target immunodominant strain-specific holes in the glycan shield of Env and are therefore unable to neutralize heterologous viruses. Although strain-specific glycan holes can be considered as a ‘‘dead-end’’ for NAb responses, they can also be exploited as an ‘‘opportunity’’ to guide autologous NAb responses towards breadth. Many bNAbs are known to interact with *N*‐linked glycans on Env [80] and there are studies suggesting that the development of bNAb lineages VRC01 and PGT121 may have been initiated by viruses lacking conserved glycans at positions 276 [81] and 137 [82], respectively. This data implies that glycan-deleted immunogens may be useful to prime the immune response against a specific site of interest. Further immunizations using immunogens in which removed glycans are gradually restored might help the mAbs to tolerate the dense glycan shield of Env and develop neutralization breadth. Thus, to drive autologous responses towards heterologous NAbs, careful selection of HIV-1 Env immunogens and immunization strategies are required to guide the humoral response into the right direction (Fig. 1).

### Focusing on conserved epitopes

One strategy to drive the antibody response towards the more conserved epitopes on Env is to restrain the exposure of off-target non-NAb epitopes such as the V3 loop and the base of soluble native-like trimers. Off-target epitopes may hamper the elicitation of bNAbs due to the hierarchy in immunodominance between non-NAb and (b)NAb epitopes [83]. B cells with undesired and high affinity non-NAbs may enter germinal centers and block antigen binding to lower affinity B cell clones that have the potential to develop into bNAbs [84, 85]. In addition, B cells with high affinity non-NAbs will take up and present more antigen, receive more help from T follicular helper cells and will therefore outcompete the desired B cells with neutralizing potential [83].

Similar to the native Env spike, which fluctuates between closed and more open conformations, the SOSIP trimer is conformationally flexible [47, 61, 86, 87]. These partially open trimers display various non-NAb epitopes such as the immunodominant V3 loop as well as CD4-induced and CD4bs-associated non-NAb epitopes. To limit the exposure of the V3 loop, the BG505 SOSIP trimer was stabilized in its pre-fusion state by a bNAb directed to the V1V2 region of the trimer, termed PGT145 [88]. Immunization of the BG505 SOSIP trimer in complex with the antigen-binding fragment of PGT145 reduced V3 reactivity in guinea pigs but did not improve autologous or heterologous NAb responses in these animals. Moreover, stabilized closed trimers have been designed to limit the exposure of undesired non-NAb epitopes. When injected into rabbits and macaques, lower levels of V3-directed non-NAbs were induced by the stabilized trimers without affecting the autologous Tier-2 responses [52–54]. The exposure and immunogenicity of V3 non-NAb epitopes was even further decreased by either the insertion of two glycans in the V3 loop or by introducing hydrophobic mutations in the V3 region [55, 89, 90]. However, restricting the V3-loop antigenicity did not improve heterologous Tier-2 responses [54]. This suggests that the elimination of a single immunodominant non-NAb epitope on Env may not be sufficient to re-direct the antibody response towards bNAb epitopes.

Soluble native-like trimers expose a region at the base of the trimer which is normally occluded by the viral membrane. The base of the trimer appears to be immunodominant in mice [91], rabbits [55, 92] and macaques [55] and mAbs elicited against this region are generally non-neutralizing since this epitope does not exist on membrane-embedded Env. The addition of glycans within and around the base of native-like trimers was able to block binding of these base-directed mAbs [55]. However, whether glycan masking also makes the base of the trimer less immunodominant in vivo still needs to be studied. A different strategy to mask the base of the trimer could be the presentation of trimers on nanoparticles to reduce off-target responses and increase autologous and heterologous NAb responses.

Another approach to develop antibody breadth is to concentrate the antibody response on conserved epitopes on Env such as the CD4bs or the fusion peptide. For the CD4bs, this can be achieved by creating holes in the glycan shield of Env to allow easier access for the mAbs to the underlying protein epitope and use these glycan holes here as an ‘‘opportunity’’ to drive NAb responses towards the CD4bs. The selective removal of glycans proximal to the CD4bs resulted in the induction of CD4bs-specific autologous NAb responses in guinea pigs and rhesus macaques [57]. These responses were, however, unable to neutralize the wild-type viral variants in which the glycans were present but it shows that it is possible to direct the antibody response towards a specific site of interest by creating an immunodominant glycan hole. Since CD4-specific bNAbs isolated from HIV-1 infected individuals possess several unusual characteristics, optimized immunogen regimens are most likely needed, including the selective removal of glycans, to select for these rare mAbs followed by immunogens in which these glycans are gradually restored in order to develop antibody breadth.

Another attractive target for HIV-1 immunogen design is the Env fusion peptide, a critical component in viral entry as well as a target for bNAbs [93–95]. In a recent study, immunization with fusion peptide coupled to carrier proteins, followed by immunization with BG505 SOSIP trimers induced cross-clade Tier-2 NAbs in the majority of immunized mice [96]. Similar responses were observed in rhesus macaques and guinea pigs although only in a subset of the animals [96]. These data indicate that vaccines designed to focus on a conserved epitope, such as the fusion peptide, can increase the breadth of the immune response.

### Overcoming viral diversity

To overcome the viral diversity of HIV-1, multiple distinct Env antigens are presumably needed to guide the humoral response towards neutralization breadth. However, the stimulation by multiple antigen variants and the influence on antibody affinity maturation is not well understood. Computational stimulations of a stochastic dynamic model of affinity maturation demonstrated that sequential immunization of antigen variants is favored over simultaneously administering a cocktail of those same antigens in eliciting bNAbs [97, 98]. However, two other computational models of affinity maturation suggest a role for immunizations with optimally designed antigen cocktails to induce breadth [99, 100]. To test this in vivo, SOSIP trimers from clades A, B and C were administered to rabbits either individually, sequentially or in a cocktail to evaluate the difference in efficacy between these strategies in eliciting NAbs [62, 101]. Lower autologous NAb responses were generally generated in rabbits immunized with a combination of trimers from different clades when compared to monovalent immunizations [101]. Moreover, sequential immunization regimes did not increase heterologous or autologous NAb titers [62, 101]. In these studies, trimers from clades A, B and C were used which were most likely too genetically distinct to drive B cell maturation towards breadth. Therefore, sequential or combinatorial regimes based on more closely related *env* sequences are most likely needed to mimic the affinity maturation process that occurs during natural infection.

Another strategy to overcome the global HIV-1 diversity is the use of mosaic antigens. These antigens are computationally optimized to have a high proportion of immunological epitopes for improved coverage of the genetic diversity of HIV-1. In one study, guinea pigs were immunized with a clade A, B, C mosaic Env gp140 but only weak and sporadic autologous Tier-2 responses were elicited [102]. In a different study, adenovirus and poxvirus vector-based vaccines expressing HIV-1 mosaic antigens of Env, Gag and Pol demonstrated substantial protection against SHIV_SF162P3_ in rhesus macaques [103]. Low levels of NAbs against SHIV_SF162P3_ were detected but NAbs against Tier-1 virus SF162 (Tier-1), Env-specific binding and non-neutralizing antibody-dependent cellular phagocytosis responses were correlated with protection [103]. As a follow-up study, both non-human primates and adults were primed with an adenovirus serotype 26 (Ad26)-based mosaic HIV-1 vaccine and boosted with either an Ad26 or Modified Vaccinia Ankara (MVA) vector with a clade C Env gp140 [104]. Both immunization regimes induced Env-specific binding antibody responses, T cell responses and antibody-dependent cellular phagocytosis in non-human primates and humans. Moreover, these three parameters correlated with protection against repeated SHIV_SF162P3_ challenges in non-human primates, but no Tier-2 NAbs were elicited.

A different approach to deal with the viral diversity of HIV-1 is the use of consensus Env immunogens which are based on HIV-1 sequences specifically designed to minimize the genetic distance between the circulating HIV-1 viruses. In one of these studies an oligomeric gp140 protein based on a group M consensus *env* gene (Con-S) was used to immunize guinea pigs and induced weakly cross-subtype NAbs against a subset of Tier-2 viruses [72, 73]. When tested as a Con-S Env gp120 in macaques similar responses were observed [74]. Although weak and sporadic, these heterologous NAb responses suggest that using a consensus M *env* gene may have potential to induce heterologous responses and should be further exploited. Future studies will have to point out whether other mosaic or consensus Env immunogens are better at eliciting bNAb responses compared to immunogens consisting of natural sequences.

### Mimicking natural infection

Vaccine strategies using a combination of highly diverse viral isolates have only elicited autologous responses, as described above [62, 101]. However, during the affinity maturation process of bNAbs in infected individuals, B cells are exposed to antigen variants that are more closely related. Longitudinal studies of HIV-1 T/F viruses and the co-evolving HIV-1 bNAb lineages have demonstrated that development of neutralization breadth relies on increasing viral Env diversity [17, 18, 23, 105]. One approach to recapitulate this co-evolutionary process is based on HIV-1 bNAbs and the natural longitudinal *env* sequences that have elicited them [17, 18], termed lineage immunogens.

In one antibody-virus co-evolution study, two different CD4bs bNAbs lineages (CH103 and CH235) were isolated from a HIV‐1 infected individual CH505 [17, 106]. To elicit CH103 lineage-like mAbs, rhesus macaques were immunized with longitudinal CH505 Envs but the majority of the animals failed to generate autologous or heterologous Tier-2 neutralization [76, 107]. Only one out of eight immunized rhesus macaques developed NAbs against the autologous CH505 Tier-2 virus and various heterologous Tier-2 viruses [76]. Plasma neutralization activity was V1V2-glycan orientated and did not target the CD4bs. Similarly, V1V2-glycan but not CD4bs targeting NAbs were elicited in experiments with germline CH103 heavy-chain-only knock-in mice. These mice are subject to gene editing and rearrangements in their antibody repertoire which may explain why they developed NAbs against the V1/V2 glycan epitope and not the CD4bs [76]. Overall, these results demonstrate that eliciting CD4bs bNAbs remains difficult even with an Env that elicited such responses during natural infection. However, when rhesus macaques were immunized with a vectored-immunogen expressing CH505 T/F Envs, NAbs were indeed elicited against the CD4bs of the T/F virus [108]. A set of six CH505 Env immunogens have now been optimized and predicted to elicit both CH103 and CH235-like bNAbs lineages [105, 109], and clinical studies are underway to test whether this will be the case.

Approximately 1% of HIV-1 infected individuals generate NAb responses with remarkable breadth and potency against most viral subtypes [110]. These individuals are described as “elite neutralizers” and demonstrate that the human B cell repertoire can overcome the extreme diversity of the circulating HIV-1 strains worldwide. Understanding why these exceptionally broad and potent responses only develop in these individuals, and more specifically which *env* sequences have induced these responses, can assist in the design of Env vaccines to elicit similar types of bNAbs. From one elite neutralizer CAP256, the V1V2-directed VRC26 lineage mAbs were isolated over time and the key viral events responsible for the development of neutralization breadth were determined [18, 23]. Based on these results, immunization strategies to elicit VRC26 lineage-like mAbs with longitudinally CAP256 Envs have been proposed using a prime to select for mAbs with long CDRH3s followed by three sequential boosts with various Envs that drove bNAb development in the CAP256 patient [23, 111].

### Targeting bNAb B cell precursors

The majority of available recombinant Env proteins or HIV-1 isolates are incapable of interacting with the inferred germline (iGL) precursors of known bNAbs [112–115]. Research efforts have been made to specifically engineer HIV-1 immunogens with increased affinity to the iGL precursors of known bNAbs, in particular to elicit CD4bs bNAb lineages [116–120]. This “germline-targeting” strategy aims to activate B cells expressing the unmutated precursor of a bNAb followed by further immunizations to guide the affinity maturation pathway towards the development of bNAbs. As the CD4bs is relatively well conserved among HIV-1 strains and because it is targeted in many HIV-1 infected individuals using genetically and structurally similar mAbs [6, 17, 121–123], it is an attractive target for germline-targeting vaccines. In particular one class of CD4bs bNAbs, the VRC01-like bNAbs, is an appealing target because of the breadth and potency of its members. However, these bNAbs display several features such as a high degree of affinity maturation [124] and an unusually short (five-residue) light chain CDR3 [125] which might be hard to elicit by vaccination. Interestingly, a new CD4bs mAb, IOMA, was recently isolated which might be easier to elicit due to its relatively low rate of somatic hypermutation and normal-length light chain CDR3 [6]. Most of the CD4bs germline-targeting immunogens are based on gp120 monomers such as the engineered outer domain (eOD)-GT6/8 and 426c.TM4ΔV1-V3 gp120‐core proteins and generally involve deletions of glycan sites and/or variable loops [116–119, 126]. When injected into knock-in mice engineered to express the iGL precursors of VRC01 or 3BNC60, these immunogens activated VRC01-class antibody responses better than regular recombinant Envs but neither the serum nor isolated mAbs possessed neutralizing activity [118, 127, 128]. Furthermore, priming with germline-targeting immunogens followed by sequential immunizations of various recombinant Envs, in which these glycans sites were gradually restored, generated NAb responses against HIV-1 isolates lacking a potential *N*-linked glycosylation site at position N276 but not when the N276 glycan was present [129, 130]. As the N276 glycan is present in the majority of HIV-1 isolates, further research should point out if CD4bs-directed mAbs can be elicited that can tolerate the N276 glycan.

While these immunogens explicitly target iGL precursors of VRC01-like bNAbs, others have developed germline-targeting immunogens to elicit bNAbs targeting different epitopes on Env. Germline-targeting immunogens based on the BG505 SOSIP trimer, designed to specifically elicit PGT121-like responses, induced cross-clade neutralization against heterologous Tier-2 viruses in the iGL PGT121 knock-in mice [77, 131]. In another study various SOSIP trimers with a glycan hole around the V2 apex were able to elicit V2-apex-focused autologous NAb responses in rabbits [78]. Interestingly, one rabbit developed NAb responses against five other heterologous Tier-2 viruses when four V2-apex targeting SOSIPS were given as a cocktail. Finally, a BG505 SOSIP trimer has been designed with binding properties to various iGL precursors of CD4bs as well as apex targeting bNAbs [120], termed BG505 SOSIP-GT1. Immunization studies in knock-in mice engineered to express the iGL precursors of VRC01 demonstrated that naive B cells with the right specificities were activated [120].

Although these data demonstrate that iGL B cell precursors of bNAbs can be activated in vivo, it is important to note that the exact germline sequences that bNAbs are derived from are generally unknown. As a result, many of the iGL precursors of bNAbs possess the CDRH3 of the mature antibody and may therefore not accurately represent the naïve B cell precursors of bNAbs found in the human antibody repertoire. This is especially a problem for germline-targeting strategies aiming to elicit bNAbs that recognize Env completely through their CDRH3.

The iGL knock-in mice models used to assess the immunogenicity of the germline-targeting immunogens also contain the pre-fixed CDRH3 of mature bNAbs. Moreover, in these knock-in mice models the majority of the B cells express the heavy-chain and/or light chain of the iGL precursors of the desired bNAb. In humans, however, only a fraction of the B cell population expresses the desired B cell receptor and these B cells will need to compete for binding to Env with a majority of non-neutralizing B cells. Future studies in humans will have to reveal whether germline-targeting immunogens, based on the binding to these artificially reverted germline sequences, can activate B cell precursors with the right specificities.

## Conclusions

With the development of native-like trimers and optimal immunization regimes, autologous Tier-2 NAb responses are now consistently elicited in different animal models. However, these NAb responses predominantly target immunodominant holes in the glycan shield of Env due to the lack of glycosylation sites that are normally present in the majority of circulating HIV-1 strains. In many cases, these strain-specific glycan holes have led to a ‘‘dead-end’’ for the development of bNAbs and efforts to reduce off-target epitopes on Env have been insufficient to focus the immune system towards more conserved epitopes and induce neutralization breadth. Therefore, glycan holes created by the selective removal of glycans near bNAb epitopes followed by gradually restoring them could serve as an ‘‘opportunity’’ to help the mAbs to tolerate the glycan shield of HIV-1 and focus the antibody response on conserved sites, such as the CD4bs and fusion peptide. Furthermore, co-evolution studies of bNAbs and virus have illuminated the complex nature of bNAb ontogeny, providing the field with valuable information on how to try to mimic this. One promising avenue is the germline-targeting strategy, with proof-of-concept experiments demonstrating that B cell precursors with the right specificities can be activated in vivo. However, to recapitulate natural infection by vaccination it most likely requires the integration of all different strategies reviewed here into one coordinated vaccination regime to efficiently induce heterologous NAbs. For example priming with germline targeting immunogens based on *env* sequences from patients that developed bNAb responses and boosting with lineage based immunogens from the same patient and/or immunogens representing consensus sequences. To determine the best route towards eliciting bNAb responses important knowledge gaps still need to be addressed. Current studies in humans will provide the field with valuable information as well as new insights how to proceed further which will hopefully lead to a vaccine that can protect against HIV-1.
